# Comparative DNA methylome analysis of estrus ewes reveals the complex regulatory pathways of sheep fecundity

**DOI:** 10.1186/s12958-020-00633-9

**Published:** 2020-08-04

**Authors:** Xiangyang Miao, Qingmiao Luo, Lingli Xie, Huijing Zhao, Xiaoyu Qin

**Affiliations:** State Key Laboratory of Animal Nutrition, Institute of Animal Sciences, Chinese Academy of Agricultural Sciences, Beijing, 100193 China

**Keywords:** Sheep, Epigenetics, Ovary, Methylation, Fecundity

## Abstract

**Background/aims:**

Sheep are important livestock with variant ovulation rate and fertility. Dorset sheep is a typical breed with low prolificacy, whereas Small Tail Han sheep with FecB mutation (HanBB) have hyperprolificacy. Our previous studies have revealed the gene expression difference between the ovaries from Dorset and HanBB sheep contributes to the difference of fecundity, however, what leads to these gene expression difference remains unclear. DNA methylation, an important epigenetic process, plays a crucial role in gene expression regulation.

**Methods:**

In the present study, we constructed a methylated DNA immunoprecipitation combined with high throughput sequencing (MeDIP-seq) strategy to investigate the differentially methylated genes between the Dorset and HanBB ovaries.

**Results:**

Our findings suggest the genes involved in immune response, branched-chain amino acid metabolism, cell growth and cell junction were differentially methylated in or around the gene body regions.

**Conclusions:**

These findings provide prospective insights on the epigenetic basis of sheep fecundity.

## Introduction

Sheep (*Ovis aries*) is an important livestock in China. One of the major challenges in sheep raising industry is how to increase the sheep fertility. Fertility depends on many factors: estrus cycle, ovulation rate and litter size, etc.; among them, the fecundity of ewe plays a central role. Ovarian folliculogenesis, is precisely regulated by the interaction of the central nervous system, the pituitary and the ovary [1, 2]. The environmental factors such as nutritional level and metabolic activity affect sheep reproduction well [3–5]. More importantly, the genetic background of ewe is in charge of its fertility. In the latest decades, a serial of studies have demonstrated the critical roles of particular genes in ovarian follicular growth. The ovine Booroola fecundity gene (FecB) was the first identified gene involved in ovulation rate and litter size, and the FecB mutant is believed to be coded by sheep bone morphogenetic receptor type 1 B (BMPR1B) gene [6, 7]. BMPR1B belongs to the TGF-beta superfamily, and several other TGF-beta family members are also thought to be associated with the sheep fertility, including growth differentiation factor 9 (GDF9) and bone morphogenetic protein 15 (BMP15) [8, 9]. These findings indicated the crucial role of genetic variations in sheep fertility.

Recent genomic studies provided a prospective view on the hereditary basis of sheep fertility. The first complete genome sequence of sheep was generated from two Texel individuals in 2014, and their findings indicated the key genes such as MOGAT2 and MOGAT3 involved in long chain fatty acid metabolism [10]. The sheep genome sequence difference could help to understand the genetic foundations of sheep fertility [11]. However, the DNA sequence is not the only thing to connect the gene and gene products. Epigenetics, how physiological phenotypic variations or environmental factors affect gene expression without gene sequence change, will also affect the sheep fertility. Typical epigenetic regulation includes non-coding RNA, chromatin variation and DNA methylation [12]. Several studies have focused on the differentially expressed microRNAs between sheep breeds with fertility variations [13–16]. In current study, we aim to explore the roles of DNA methylation in the epigenetic regulation of ewe fecundity.

Small Tail Han sheep is a widely bred sheep breed in China with year-round estrus. The Han sheep is well-known for its hyperprolificacy, the mean litter size of Han sheep is 2.61 [17]. FecB mutant was identified in most of high prolific Han sheep [18]. On the other hand, Dorset sheep is a typical sheep breed with low fecundity, the mean litter size of Dorset sheep is only 1.45 [19]. A comparison of the hereditary background of Dorset sheep and Han sheep will provide important information to get a better grasp of sheep fertility. Ovary plays the central role in ewe fecundity. Our previous study has compared the ovarian mRNA and miRNA expression difference between Han sheep and Dorset [14]. However, the differentially expressed miRNAs cannot explain the mRNA expression difference completely. In the present study, in order to get a deeper understanding of the role of ovarian epigenetic regulation in sheep fertility, we construct a methylated DNA immunoprecipitation combined with high throughput sequencing (MeDIP-seq) strategy to identify the ovarian genes with different DNA methylation level between Dorset sheep and Han sheep with FecB mutant. As we know, this is the first comparative study to investigate the DNA methylome distribution in sheep ovary. Our findings will contribute to the understanding of the epigenetic mechanisms of sheep fertility.

## Results

### Global mapping of DNA methylation in sheep ovarian genome

In the present study, we mapped the global DNA methylation status of ovarian genome collected from Dorset and Han sheep using MeDIP-seq. A total of 5.53 Gb and 5.63 Gb data were obtained from the ovarian samples of Dorset and HanBB sheep respectively, including 112,892,144 (Dorset) and 114,963,972 (Han) raw reads. Of the total reads, 93.71 and 93.88% were mapped to the sheep reference genome (ftp://ftp.ncbi.nih.gov/genomes/Ovis_aries) for Dorset and HanBB group, respectively. Among them, 55.18 and 55.82% were uniquely mapped to sheep genome, respectively.

**Fig. 1:**
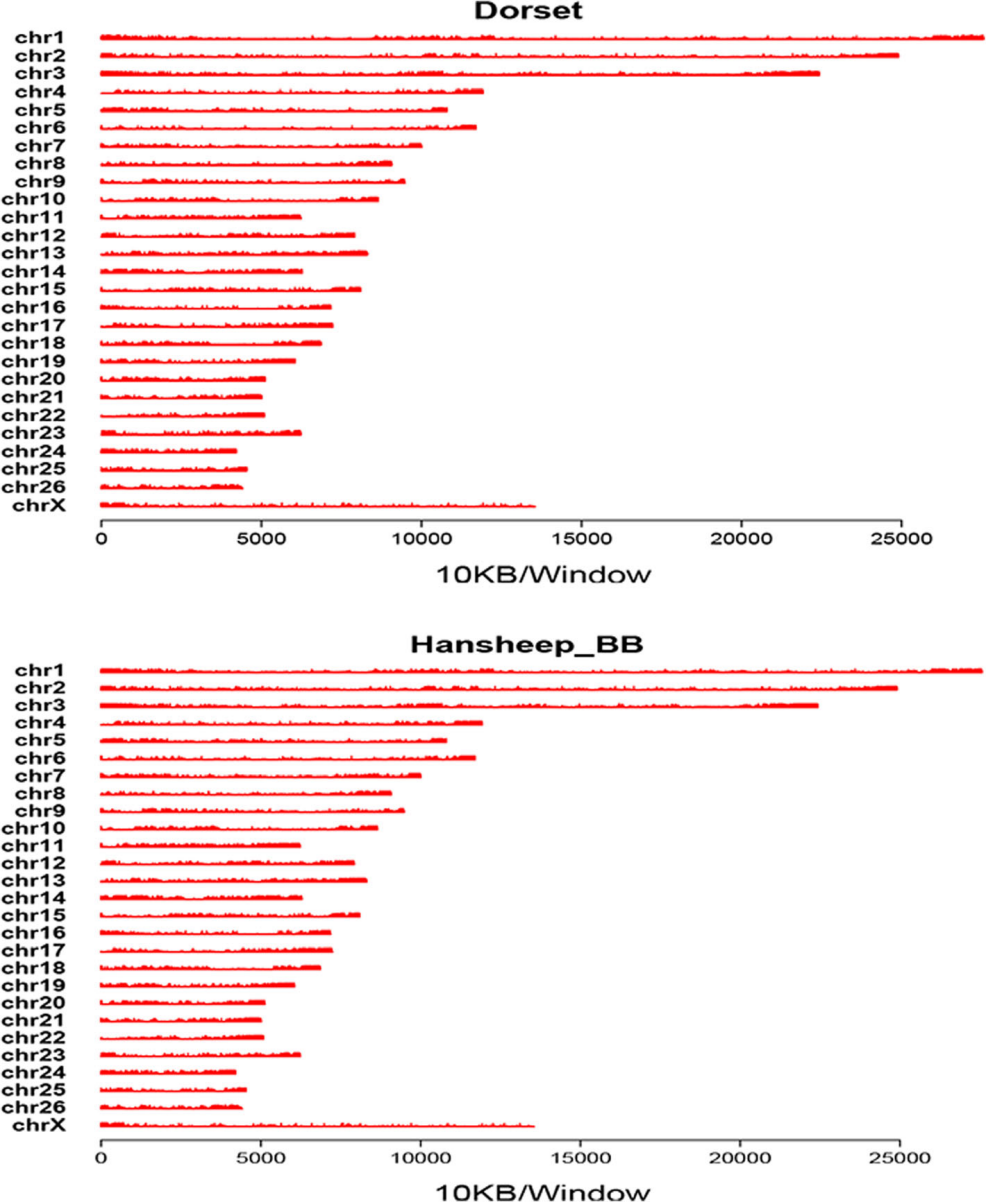
Reads distribution on chromosomes. The distribution of reads in chromosomes 1 to 26, and chromosome X of the sheep genome in Dorset and Han BB sheep are shown in red for each sample. MeDIP-seq reads were plotted in 10 kb windows along the chromosome

**Fig. 2:**
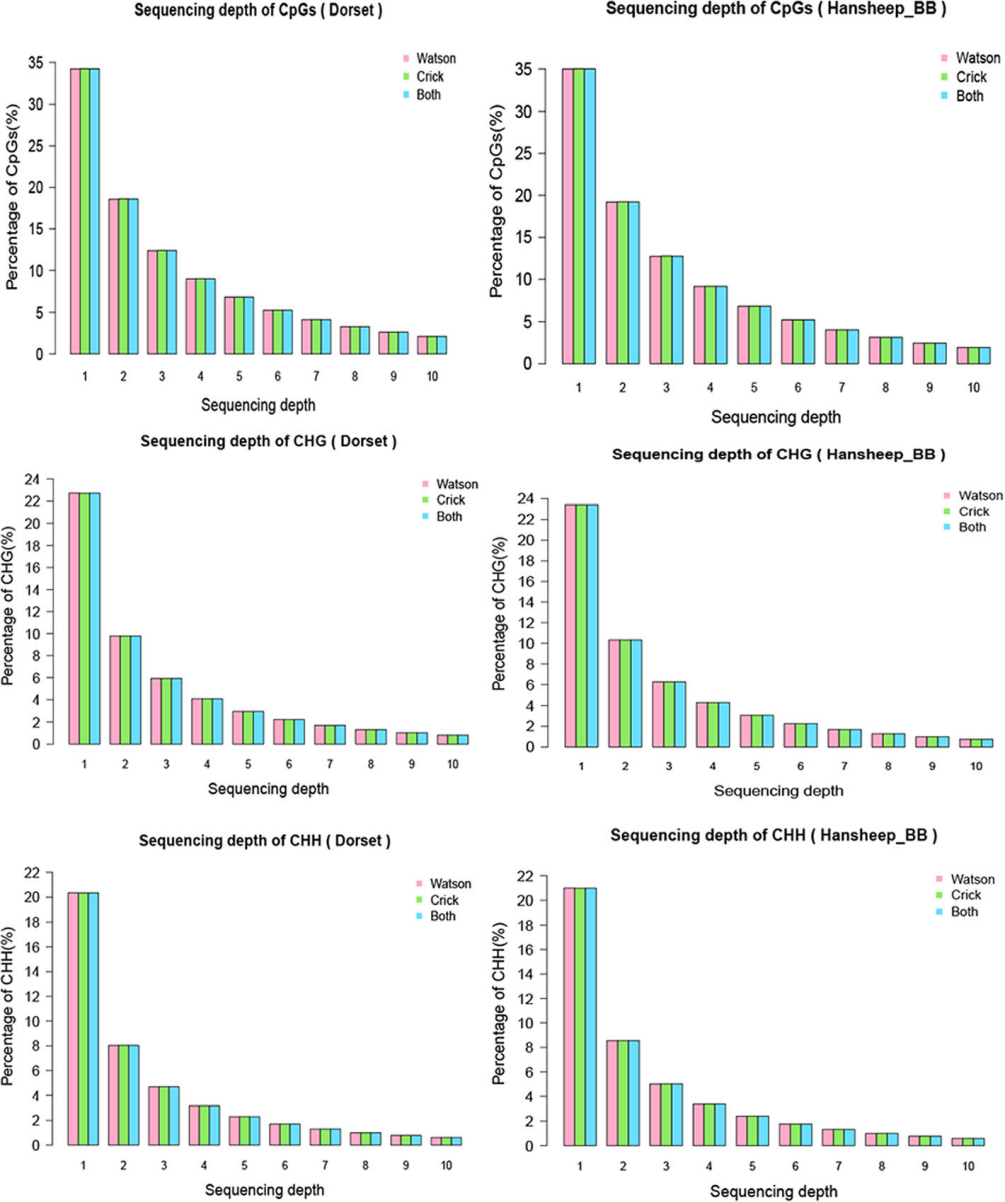
Sequencing depth of CpG, CHG and CHH. The pencentage of the sequencing coverage of CpG, CHG and CHH in Dorset and HanBB sheep genome are shown in red (Watson strand), green (Crick strand) and blue (both strand)

Uniquely mapped MeDIP-seq reads were detected in all chromosomes of ewe. (GGA1–26 and chromosome X) (Fig. 1). Genome coverage analysis illustrated that most mapped regions had low sequencing depth (1–4), and no more than 10% of mapped genome had higher sequencing depth (5–10) in both groups. Additionally, the genome coverage of the CpG sites, and non-CpG methylation such as CHG, and CHH (with H being A, C, or T)) sites were similar between two groups. Around 35% of CpG sites, and 20% of CHG or CHH sites had only one sequencing depth in both groups, and only a small number of regions had high (5–10) sequencing depth in CpG, CHG and CHH sites (Fig. 2).

**Fig. 3:**
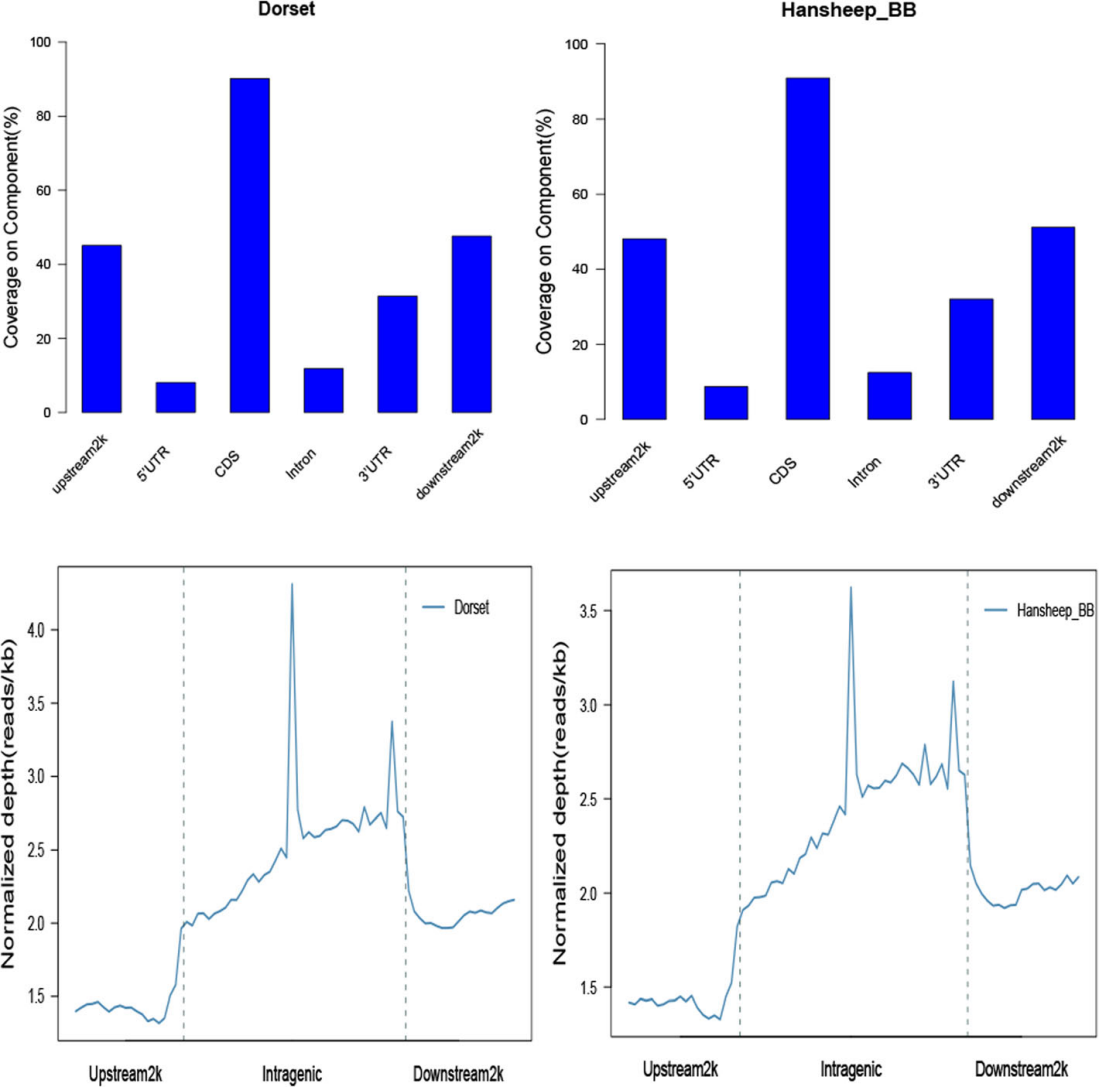
Methylated reads distribution. (A) The proportion of methylated reads were shown in the eight elements of genome of Dorset and HanBB sheep. (B) Normalized depth of the reads in the intragenic and the upstream 2 kb or the downstream 2 kb

The mapped reads distributed on different genomic regions exhibits the methylation pattern. Over 16% of uniquely mapped reads were present at the gene body regions in both groups, followed by CpG islands (CGIs, around 11%) and introns (~ 9%). Further analysis illustrated that within the intragenic elements, the DNA methylation level gradually increased at the upstream region near the transcription start sites (TSSs), peaked at the middle of intragenic region and had another smaller peak just before the transcription termination site (TTS), and dropped dramatically and remained a level of DNA methylation similar as the TSS at the downstream 2 k area (Fig. 3). Many CpG islands located in repetitive elements, and we observed different methylation levels in different repetitive elements, with high methylation in LINE/RTE-BovB, LINE/L1 and SINE/BovA.

### DNA methylation profile in sheep genome

**Fig. 4:**
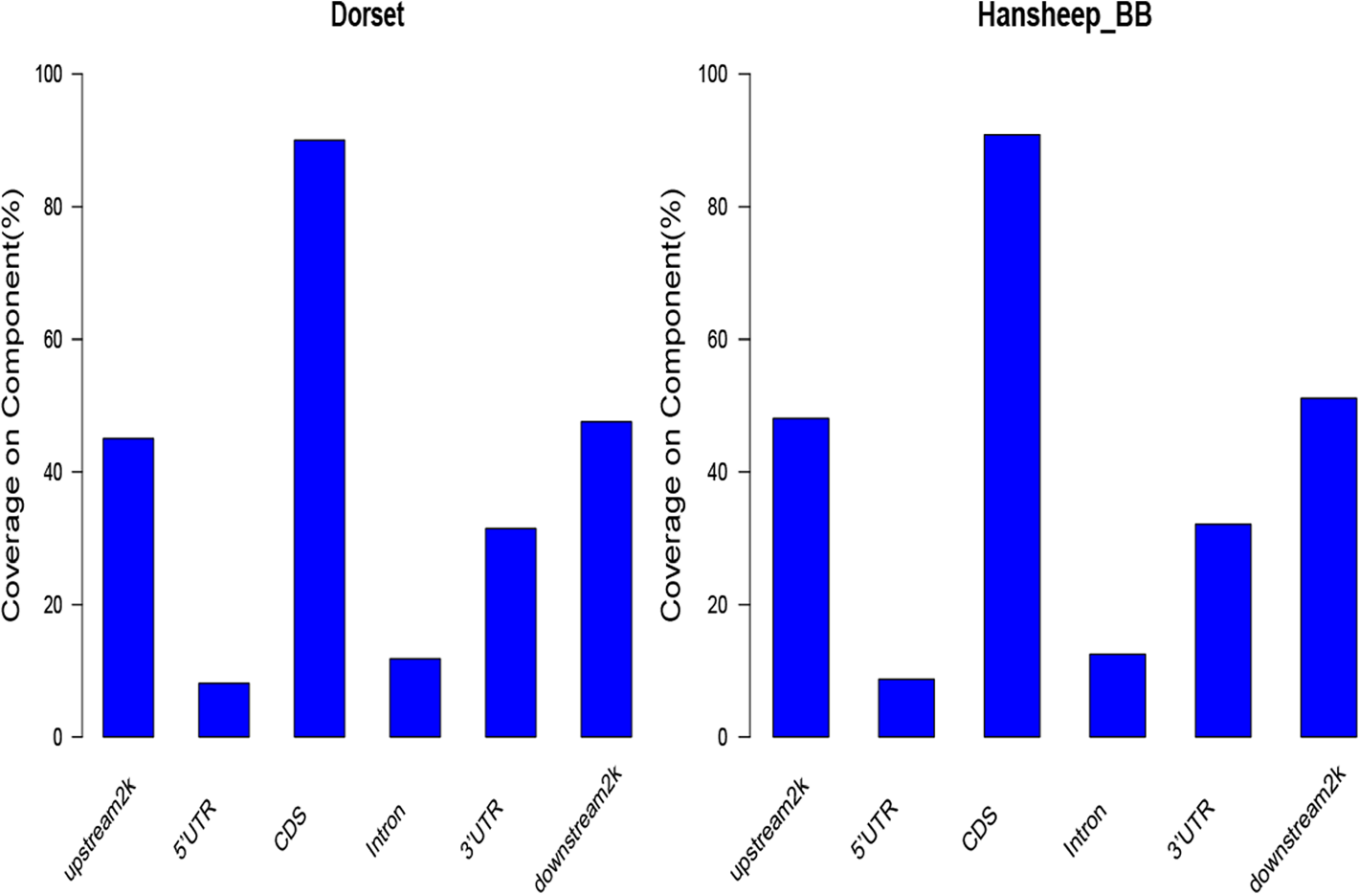
Methylated peaks coverage in different gene elements. The coverage of methylated peaks in the intragenic (5’UTR, CDS, intron, 3’UTR) and the upstream 2 kb or the downstream 2 kb components of Dorset and HanBB sheep genome

To investigate the genome-wide DNA methylation profiles of sheep genome, the uniquely mapped reads were used to detect the methylated peaks, which means the areas in a genome that have been enriched with aligned reads with methylated cytosine. Using MACS1.4.0, we obtained 217,427 and 208,732 methylated peaks in Dorset and HanBB group, covering 8.60 and 9.02% of sheep genome respectively. Most of peaks had 5 to 20 CpG sites. The peak distribution in different components of the genome were then further analyzed. The majority of peaks covered the coding sequence (CDS) regions followed by 2 kb downstream of TTS region, 2 kb upstream of TSS region and 3′-UTR. Intron and 5′-UTR had much fewer peaks (Fig. 4). Here, upstream2k were regarded as the proximal promoter.

### Differential DNA methylation genes in sheep ovary

**Fig. 5:**
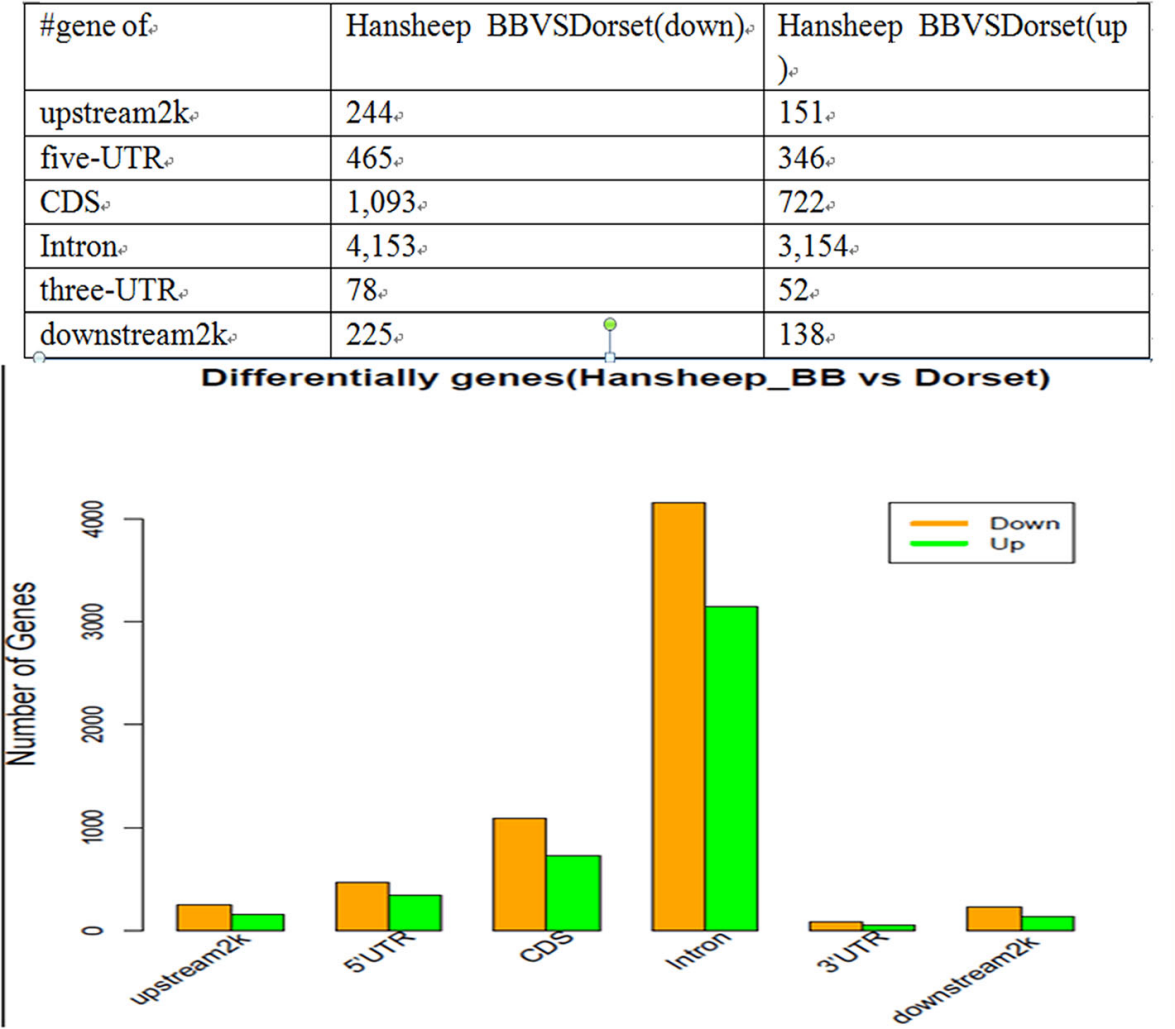
Differentially methylated genes in different gene elements. The number of identified genes with differentially methylated level in the intragenic (5’UTR, CDS, intron, 3’UTR) and the upstream 2 kb or the downstream 2 kb components between Dorset and HanBB sheep

In the present study, we compared the differentially methylated regions (DMRs) between Dorset and HanBB sheep. Genes with methylation peaks identified in either the promoter or gene body regions were considered as methylated genes. A total of 10,821 differentially methylated genes were successfully identified. Compared with Dorset sheep, in HanBB ewe ovaries, 395 differentially methylated in the 2 kb upstream of TSS region (244 down-methylated, 151up-methylated), 181 in the 5’UTR (30 down-methylated, 151 up-methylated), 1815 in the CDS (1093 down-methylated, 722 up-methylated), 7307 in the intron (4153 downmethylated, 3154 up-methylated), 130 in the 3’UTR (78 down-methylated, 52 up-methylated), and 363 2 kb downstream of TTS region (225 down-methylated, 138 up-methylated). More genes were down-methylated (6258 genes) than up-methylated (4563 genes) in HanBB group compared to Dorset group (Fig. 5, Additional file 1).

### MeDIP-seq data validation

**Fig. 6:**
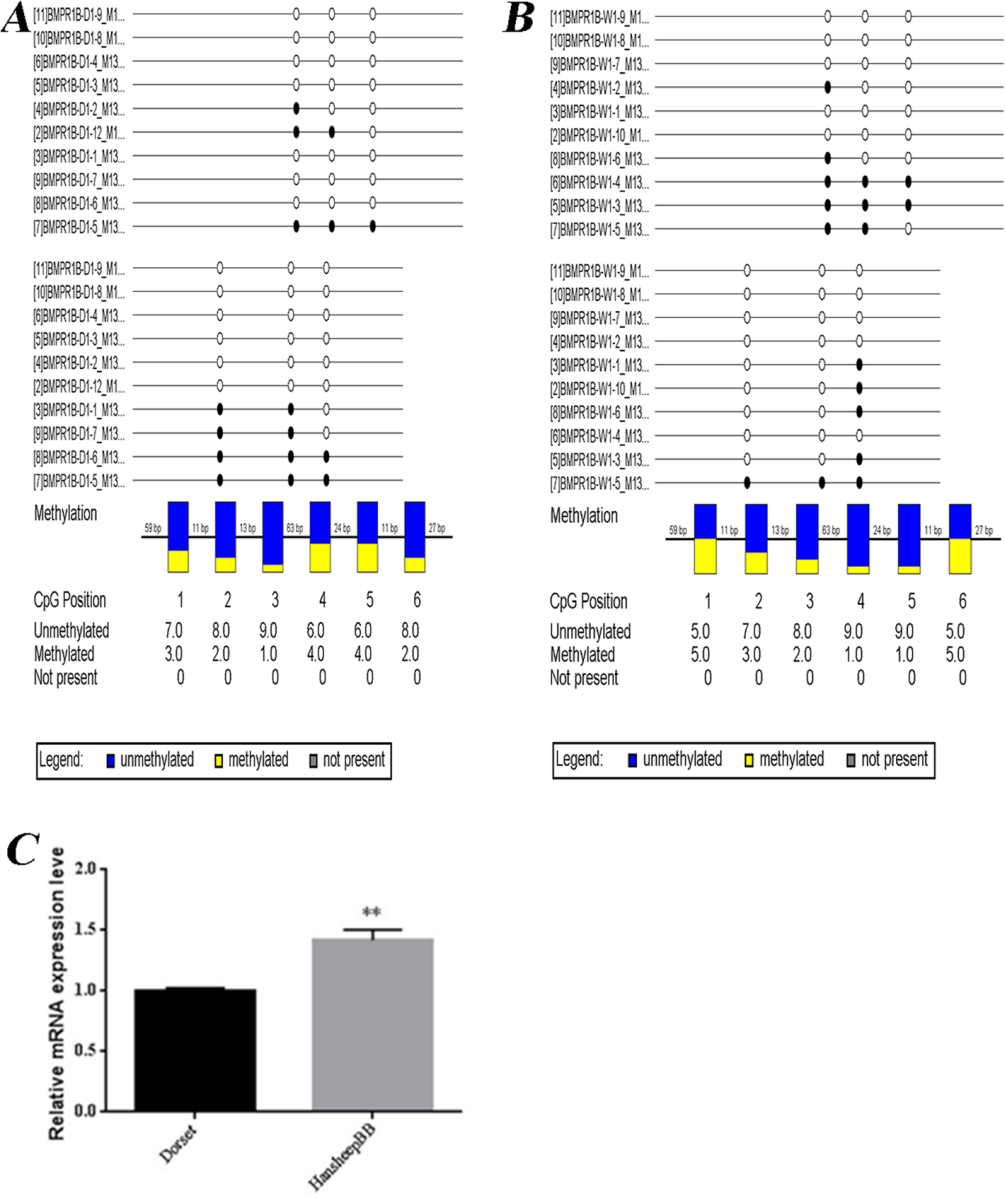
Sequencing data validation. A and B, Bisulfite Sequencing of BMPR1B in Dorset sheep (A) and HanBB sheep (B). C, RT-PCR of krit1. **, *p* < 0.01, vs. Dorset. sheep

We constructed a bisulfite sequencing strategy to validate the DMRs identified from MeDIP-seq. The result of gene BMPR1B-F from the bisulfite sequencing confirmed that the DNA methylation levels in gene BMPR1B were different between two sheep. (Fig. 6A, B).

### GO and KEGG analysis of differentially methylated genes

To identify the biological functions of identified ovarian differentially methylated genes between Dorset and HanBB sheep, we first applied GO annotation analysis. GO annotation indicated that the methylated genes involved in three terms: biological process, cellular component, and molecular function. We mainly focused on the biological process. Compared with the Dorset sheep, among the genes with DMR in upstream2k, down-methylated genes in HanBB sheep were enriched in innate immune response, and up-methylated genes were involved in monocyte chemotaxis, positive regulation of axon extension and regulation of developmental growth. In 5′-UTR elements, no genes with DMR were enriched in any processes with significance. In CDS elements, the down-methylated genes were involved in developmental cell growth, synapse assembly, collateral sprouting and activation of MAPKK activity, while the up-methylated genes were enriched in regulation of histone methylation and DNA methylation on cytosine, including the *Ovis aries* DNA (cytosine-5-)-methyltransferase 3 beta (DNMT3B), transcript variant 1–6. In intron elements, which is the major region of DMR distribution, the down-methylated genes were related to cellular protein modification process, locomotion, ion transport and cell projection organization, as well as the up-methylated genes were also connected to cellular protein modification process and ion transport, besides, other processes such as G-protein coupled receptor signaling pathway and microspike assembly were involved. In 3′-UTR, the granulocyte differentiation were the only significant enriched process of genes with DMR, which were up-methylated in HanBB sheep. And in 2 kb downstream of TTS region, down-methylated genes were related to carbohydrate catabolic process, and up-methylated genes had no significant enriched processes. Taken together, the genes with DMR were primarily involved in biological processes such as cell signaling, protein modification, ion transport and immune regulation.

To further understand the functions of genes with DMR, we performed KEGG pathway analysis to predict the potential significant pathways (q < 0.05) related to differentially methylated genes. Compared with Dorset group, in HanBB sheep, the genes with up-methylated upstream2k region were associated with valine, leucine and isoleucine biosynthesis; the down-methylated 5′-UTR genes were related to T cell receptor signaling pathway; the genes with down-methylated CDS were involved in ECM-receptor interaction, tight junction, renin-angiotensin system and toxoplasmosis, as well as the up-methylated CDS were also related to ECM-receptor interaction, besides, the pathways of amoebiasis, cysteine and methionine metabolism and protein digestion and absorption were involved. In the intron elements, both the down-methylated and up-methylated genes were connected to tight junction, vascular smooth muscle contraction and long-term potentiation significantly, besides, the down-methylated genes were involved in ErbB signaling pathway. Taken together, the genes with DMR were enriched in pathways associated with cell-cell junction.

### DNA methylation and gene expression level

In our previous study, we have explored the different transcriptome of ewe ovaries between Dorset and HanBB sheep (Additional file 2) [20]. To investigate the ovarian gene expression level in current samples, we constructed a RNA-seq strategy to detect the differentially expressed genes between Dorset and HanBB ovaries [20]. To validate the RNA-seq data, the mRNA expression level of *krit1* were measured by real-time PCR, the PCR results were consistent with the RNA-seq data, suggests the RNA-seq results were reliable (Fig. 6C).

DNA methylation plays a critical role in gene expression modification. To explore whether the differentially methylated region in or around gene body affects the gene expression, the DNA methylation patterns were co-analyzed with the gene expression profiles. A total of 63 differentially expressed genes between Dorset and HanBB sheep ovaries were identified with DMR in or around the gene body, and the major DMR of these genes were located in the intron regions (Additional file 3). GO analysis indicated that “the hydrolase activity, acting on ester bonds” was the only significant molecular function term, while the biological process and cellular component had no significant terms. No significant pathways were enriched in KEGG analysis.

## Discussion

The variation of sheep fecundity depends on the genetic background and gene expression level of ewe ovary. Our previous studies have revealed the differential ovarian gene expression between low-prolificacy Dorset sheep and high-prolificacy Small Tail Han sheep with FecB genotype [14]. FecB gene mutation leads to higher ovulation rate and litter size in sheep [18]. So the comparison between Dorset sheep and Han sheep with FecB mutation provided an excellent model to investigate the influence of genetic backgrounds of ovary on fertility. Our previous RNA-seq study have pointed out that those genes involved in cell proliferation, steroid metabolic processes, ribosome assemble and stress response may contribute to the hyperprolificacy of HanBB sheep [14]. However, what leads to the difference of these gene expression remain unclear. Epigenetics has been believed to play an important role in gene expression regulation. Epigenetics developed quickly in the latest decade, based on the development of next-generation sequencing technique [21]. Histone modification, DNA methylation and non-coding microRNAs are all possible to affect the gene expression and thereby induce physiological modification. Our previous studies have demonstrated the roles of microRNAs on sheep physiology such as adipogenesis and lipid metabolism, muscle development and reproduction [14, 22–25]. However, how the DNA methylation affects gene expression in sheep, particularly in ovary mature, ovulation and fecundity is still unknown. In the present study, we conducted a MeDIP-seq experiment to investigate the ovarian genome methylation difference between Dorset and HanBB sheep. Limited to our knowledge, this is the first study to explore the role of DNA methylome in sheep fecundity.

DNA methylation, refers to the methylated modification of 5′ position of cytosine residues in nucleotide, primarily occurred at the C base within CpG dinucleotides, the CpG rich region in genome sequence are thereby termed as CpG islands 30 years ago [26]. Recent studies found that the cytosine residues with in CHG or CHH (with H could be A, C, or T) sites could be methylated too [27]. In the current study, the genomic methylated cytosine in both CpG sites and CHG or CHH sites were measured by the technique known as MeDIP-seq. In MeDIP-seq, anti-5-methylcytosine antibody was used to recognize and pull-down the DNA fragments with methylated cytosines, and then these enriched methylated DNA fragments were sequenced by next-generation sequencing. In our present study, the sequencing reads covered all the chromosomes, suggests the MeDIP-seq experiment works well.

DNA methylation affects various crucial biological processes, such as embryonic development, cellular differentiation, tissue-specific gene expression, genomic imprinting, suppression of repetitive elements, carcinogenesis, aging, X chromosome inactivation, and chromosome stability, via regulating the gene expression and silencing [28–30]. The DNA methylation in different genomic regions have different effects on gene expression. The DNA methylation occurred in the promoter region normally leads to gene silencing via blocking the accessing and target site binding of DNA binding proteins or recruiting the methyl-CpG binding proteins to repress the gene transcription, whereas the demethylation of CpG islands in promoter region may result to gene expression [28, 31]. Additionally, the DNA methylation in the gene body often promotes gene expression, which may partly attribute to transcript splicing regulation [32]. In our current study, the distribution of all methylated reads illustrated that the gene body are the most methylated regions in sheep ovarian genome, suggests only the intragenic regions were hyper-methylated. Those genes with methylation enriched peaks in or around gene body were thought to be DNA methylation affected genes. In these genes, the highly methylated gene elements were CDS, followed by 2 kb upstream of TSS, indicates both the coding exon regions and promoters were methylated in general. Then we analyzed the genes with DMRs in or around gene body between Dorset sheep and HanBB sheep. Interestingly, in these differentially methylated genes, most of DMRs were in enriched in intron, followed by CDS. Other DMRs were mainly in 5′-UTR and upstream2k regions. The GO and KEGG annotation analysis indicates that in potential promoter regions such as upstream2k, the differentially methylated genes were mainly related to immune regulation processes such as innate immune response and monocyte chemotaxis, and the branched-chain amino acids (BCAAs) metabolism were also involved. Healthy inflammatory response improves folliculogenesis, oocyte maturation and ovulation, whereas unhealthy chronic, low-grade inflammation (metaflammation) harms ovulation [33], our findings implied that ewe’s ovulation may be regulated via DNA methylation modification. Nutrition intake such as BCAAs have important influence on sheep fertility [34], and our study implies that utilization of BCAAs may be a reason of different prolificacy between Dorset and HanBB sheep. In the gene body regions such as CDS and intron, the differentially methylated genes were related to cell growth, protein modification, ion transport, and particularly cell junction. The cell junction take a role in ovarian physiology, such as blood-follicle barrier and endothelial permeability, and affects the initiation of follicle growth [35, 36]. The different levels of gene body methylation in cell junction related proteins provides some new information about the role of cell-cell junction in sheep fecundity.

The co-analysis of RNA-seq and MeDIP-seq revealed that many genes with higher expression level in HanBB sheep had lower methylated intron level, compared with Dorset sheep, such as *krit1*. DNA demethylation in intron may lead to up-regulated gene expression via increasing the intronic enhancer activity [37]. Krit1 takes part in SOD2-dependent catabolism of intracellular reactive oxygen species [38]. Many evidences indicate that oxidative stress has harmful influence on female reproduction (reviewed in [39]), the demethylated intron-associated enhanced expression of krit1 may contribute to the high fecundity in HanBB sheep.

## Conclusion

To explore epigenetic mechanisms of the hyperprolificacy of Small Tail Han sheep with FecB genotype, we here constructed a MeDIP-seq based comparative DNA methylomics strategy to investigate the differentially methylated genes between the ovaries from HanBB and Dorset sheep, and our findings provide prospective insights on the differences of genomic DNA methylation level between hyperprolific HanBB sheep and relative low prolific Dorset sheep. The roles of DNA methylation in sheep fecundity are worthy further study.

## Methods

### Sheep sample preparation

The tissue samples used in the present study were the same as our previous study [14]. The adult Han ewes with the FecB mutation in the BMPR1B genotype BB were regarded as Han group, which has the high fecundity; meanwhile, The adult Dorset ewes were used as a control low-reproducing Dorset group. All animals were kept under similar conditions and provided water and food ad libitum. All ewes in the experiment were treated to synchronize estrus as previous studies [14]. Twenty-four hours after spontaneous estrus were detected, all ewes were euthanized and whole ovaries were excised to obtain better ovulation points on the surfaces of the ovaries. All samples were immediately snap-frozen in liquid nitrogen and stored at − 70 °C for genome DNA and ovarian RNA extraction.

### DNA extraction

Genomic DNA was extracted from the ovaries with the use of a phenol-chloroform extraction method, and DNA quality was evaluated by agarose gel electrophoresis and spectrophotometer. Genomic DNA isolated from the three Han sheep with FecB mutant were mixed in equal amounts to generate a pooled sample as high prolific Han sample, and genomic DNA isolated from the three Dorset sheep were mixed to generate the low prolific Dorset sample.

### MeDIP-seq analysis

Genomic DNA mixtures were sonicated to gain fragments in the range 100–500 bp, and dAs were added to the 3′ ends of the DNA fragments. The adaptors were then ligated to the two ends of each fragment with paired-end DNA sample prep kit (Illumina) according to the manufacturer’s protocol. The double-stranded DNA was denatured to single strands, and then the anti-5-methylcytosine mouse monoclonal antibody (Calbiochem) was used to immunoprecipitate the DNA fragments containing regions of methylated cytosines with magnetic methylated DNA immunoprecipitation kit (Diagenode). The precipitated DNA fragments were then amplified by PCR and separated by agarose gel electrophoresis. The 200–300 bp fragments were excised and extracted using a gel extraction kit (28,706; Qiagen). The selected DNA fragments were quantified using an Agilent 2100 Analyzer. The quantified library was sequenced in an Illumina HiSeq 2000 at the BGI (Shenzhen, China).

### MeDIP-seq sequence alignments and data analyses

Quality control of raw data from sequencing was performed using FastQC (http://www.bioinformatics.babraham.ac.uk/projects/fastqc/). The reads containing adapter, or containing over 10% of N nucleotides, or over 50% of contained nucleotides had quality value Q < 21 were removed from the raw data, and all left reads were saved as clean reads in FASTQ files. The clean data were then aligned to the sheep reference genome (Ovis_aries, ftp://ftp.ncbi.nih.gov/genomes/Ovis_aries) with SOAPaligner v 2.21 (http://soap.genomics.org.cn/) with no more than 2 bp mismatches. The reads distribution in sheep chromosomes and in the different genome components including upstream2k, 5′-untranslated region (UTR), 3′-UTR, CDS, introns, CpG islands (CGIs), downstream2k, and repeats were analyzed; as well as the genome coverage of the CG, CHG, and CHH sites under different sequencing depths and distributions of MeDIP-Seq reads in different CG density regions.

Methylation peaks (known as genome-wide highly methylated regions: regions with sequencing tags more than 20, and a *p* value < 1 × 10^− 5^) scanning was conducted with MACS 1.4.0 (http://liulab.dfci.harvard.edu/MACS/) [40]. The distribution of peaks in different components of sheep genome (upstream2k, 5’UTR, CDS, Intron, 3’UTR, and downstream2k) were analyzed. To identify the differentially methylated peaks between Dorset and Han group, peak regions were assembled and the number of reads of each sample was calculated. The numbers of reads in each peak were assessed using chi-square, and false discovery rate (FDR) statistical method was used to avoid false positive data. *p* ≤ 0.01 was considered significant. Only those peaks with reads covered in the same genomic components from both groups, and the difference in reads numbers between two groups greater than twofold with significance were identified as differentially methylated peaks. All genes within differentially methylated peaks were annotated using gene ontology (GO) analysis and Kyoto Encyclopedia of Genes and Genomes (KEGG) pathway analysis. The terms and pathways with FDR < 0.05 were thought to be significant.

### RNA-seq and real-time polymerase chain reaction (PCR) validation of differentially expressed genes (DEGs)

RNA-seq data used in the present analysis was published in our previous study. To verify the DEGs identified in RNA-seq, real-time PCR was applied, the procedure was similar as previous study [14]. Total RNA was extracted from each ovaries by using TRIZOL reagent (Invitrogen, Carlsbad, CA, USA) and reverse transcription was conducted using Thermo First cDNA Synthesis Kit (SinoGene) according to manufacturer instructions. The total RNA (1 μg) from each ovary sample was retrotranscribed within the presence of oligodeoxythymidylic acid primers. Real-time PCR was performed using the StepOnePLUS (Applied Biosystems). Each qPCR sample was run in a 15-μl total volume with SG Green qPCR Mix (SinoGene). The following thermocycling conditions were used: 10 min at 95 °C and 45 cycles of 95 °C for 15 s and 60 °C for 15 s, followed by a melting ramp from 60 to 95 °C, holding for 45 s on the first step (60 °C), followed by 15 s holds at 95 °C. All reactions were performed in triplicates. GAPDH was used as the internal control. The data from the real-time PCR was analyzed by the 2^–ΔΔCT^ method [41]. The primer for gene *krit1* was as followed, Forward: 5′- CAGAATGGATGGATCATACCG-3′, Reverse: 5′- CTGCGTTTCTTGAGAGAGAC-3′.

### Bisulfite sequencing

To validate the MeDIP-seq data, the gene BMPR1B-F was selected for bisulfite sequencing. 500μg of individual DNA sample from each group was treated with the EZ DNA Methylation-Gold Kit (ZYMO) and then incubated at 95 °C for 10 min and 2.5 h at 64 °C for bisulfite conversion. After the reaction, the modified DNA samples were extracted for bisulfite sequencing PCR, and the forward primer: 5′- ATAGGGTTGTTGGGTAGTGAGTT-3′, and reverse primer: 5′- AATCTTTCTTTCCCTACCCTCTAT-3′. The PCR were performed in a 25 μL reaction mixtures that contain 2 μL modified DNA template, 1 μL primers for forward and reverse strand each, 0.15 μL Taq enzyme, 2.5 μL 10X PCR buffer, 1 μL Mg^2+^ (50 mM), 0.5 μL dNTP (10 mM) and 17.85 μL ddH2O with the following program: 94 °C for 2 min, followed by 10 cycles of denaturation at 94 °C for 30 s, annealing at 60 °C to 50 °C Δ1°C temperature ladder for 30 s, extension at 72 °C for 30 s, and then followed by 30 cycles of 94 °C for 30 s, 50 °C for 30 s, 72 °C for 30 s, and a final extension at 72 °C for 5 min. The PCR products were purified and subcloned into *pEASY* - T1 vector (transgen) for sequencing.

### Statistical analysis

All data are presented as the mean ± SD. The significance of differences in data between the groups was determined by one-way ANOVA analysis of variance followed by the student’s t-test for equality of variances using SPSS 17.0 (IBM, USA). Differences were considered statistically significant at *p* < 0.05.

## Supplementary information

**Additional file 1.**

**Additional file 2.**

**Additional file 3.**

## Data Availability

The accession number GSE107829 for DNA-Seq data. https://www.ncbi.nlm.nih.gov/geo/query/acc.cgi?acc=GSE107829. Theaccession number GSE107935 for RNA-Seq data. https://www.ncbi.nlm.nih.gov/geo/query/acc.cgi?acc=GSE107935.

## References

[1] Evans AC (2003). Ovarian follicle growth and consequences for fertility in sheep. Anim Reprod Sci.

[2] McNeilly AS (1991). The ovarian follicle and fertility. J Steroid Biochem Mol Biol.

[3] Gulliver CE, Friend MA, King BJ, Clayton EH (2012). The role of omega-3 polyunsaturated fatty acids in reproduction of sheep and cattle. Anim Reprod Sci.

[4] Padmanabhan V, Veiga-Lopez A (2014). Reproduction symposium: developmental programming of reproductive and metabolic health. J Anim Sci.

[5] Boland MP, Lonergan P, O'Callaghan D (2001). Effect of nutrition on endocrine parameters, ovarian physiology, and oocyte and embryo development. Theriogenology.

[6] Souza CJ, MacDougall C, MacDougall C, Campbell BK, McNeilly AS, Baird DT (2001). The Booroola (FecB) phenotype is associated with a mutation in the bone morphogenetic receptor type 1 B (BMPR1B) gene. J Endocrinol.

[7] Montgomery GW, Crawford AM, Penty JM, Dodds KG, Ede AJ, Henry HM, Pierson CA, Lord EA, Galloway SM, Schmack AE (1993). The ovine Booroola fecundity gene (FecB) is linked to markers from a region of human chromosome 4q. Nat Genet.

[8] Juengel JL, Bodensteiner KJ, Heath DA, Hudson NL, Moeller CL, Smith P, Galloway SM, Davis GH, Sawyer HR, McNatty KP (2004). Physiology of GDF9 and BMP15 signalling molecules. Anim Reprod Sci.

[9] Persani L, Rossetti R, Di Pasqual E, Cacciatore C, Fabre S (2014). The fundamental role of bone morphogenetic protein 15 in ovarian function and its involvement in female fertility disorders. Hum Reprod Update.

[10] Jiang Y, Xie M, Chen W, Talbot R, Maddox JF, Faraut T, Wu C, Muzny DM, Li Y, Zhang W, Stanton JA, Brauning R, Barris WC (2014). The sheep genome illuminates biology of the rumen and lipid metabolism. Science.

[11] Cockett NE (2006). The sheep genome. Genome Dyn.

[12] Goldberg AD, Allis CD, Bernstein E (2007). Epigenetics: a landscape takes shape. Cell.

[13] Shen H, Chen HY, Jia B, Han GH, Zhang YS, Zeng XC (2015). Characterization and expression analysis of microRNAs in Qira black sheep and Hetian sheep ovaries using Solexa sequencing. Genet Mol Res.

[14] Miao X, Luo Q, Qin X (2015). Genome-wide transcriptome analysis of mRNAs and microRNAs in Dorset and small tail Han sheep to explore the regulation of fecundity. Mol Cell Endocrinol.

[15] Miao X, Luo Q, Zhao H, Qin X (2016). Ovarian transcriptomic study reveals the differential regulation of miRNAs and lncRNAs related to fecundity in different sheep. Sci Rep.

[16] Miao X, Luo Q, Zhao H, Qin X (2016). Genome-wide analysis of miRNAs in the ovaries of Jining Grey and Laiwu black goats to explore the regulation of fecundity. Sci Rep.

[17] Tu YR, Tu YR (1989). Small tailed Han sheep. The sheep and goat breeds in China.

[18] Hua GH, Yang LG (2009). A review of research progress of FecB gene in Chinese breeds of sheep. Anim Reprod Sci.

[19] Casas E, Freking BA, Leymaster KA (2004). Evaluation of Dorset, Finnsheep, Romanov, Texel, and Montadale breeds of sheep: II. Reproduction of F1 ewes in fall mating seasons. J Anim Sci.

[20] Miao X, Luo Q, Zhao H, Qin X (2016). Co-expression analysis and identification of fecundity-related long non-coding RNAs in sheep ovaries. Sci Rep.

[21] Sarda S, Hannenhalli S (2014). Next-generation sequencing and epigenomics research: a hammer in search of nails. Genomics Inform.

[22] Miao X, Luo Q, Qin X, Guo Y, Zhao H (2015). Genome-wide mRNA-seq profiling reveals predominant down-regulation of lipid metabolic processes in adipose tissues of small tail Han than Dorset sheep. Biochem Biophys Res Commun.

[23] Miao X, Luo Q, Qin X, Guo Y (2015). Genome-wide analysis of microRNAs identifies the lipid metabolism pathway to be a defining factor in adipose tissue from different sheep. Sci Rep.

[24] Miao X, Luo Q, Qin X (2015). Genome-wide analysis reveals the differential regulations of mRNAs and miRNAs in Dorset and small tail Han sheep muscles. Gene.

[25] Miao X, Luo Q (2013). Genome-wide transcriptome analysis between small-tail Han sheep and the Surabaya fur sheep using high-throughput RNA sequencing. Reproduction.

[26] Bird AP (1986). CpG-rich islands and the function of DNA methylation. Nature.

[27] Cokus SJ, Feng S, Zhang X, Chen Z, Merriman B, Haudenschild CD, Pradhan S, Nelson SF, Pellegrini M, Jacobsen SE (2008). Shotgun bisulphite sequencing of the Arabidopsis genome reveals DNA methylation patterning. Nature.

[28] Bernstein BE, Meissner A, Lander ES (2007). The mammalian epigenome. Cell.

[29] Li E, Beard C, Jaenisch R (1993). Role for DNA methylation in genomic imprinting. Nature.

[30] Jones PA, Takai D (2001). The role of DNA methylation in mammalian epigenetics. Science.

[31] Bird A (2002). DNA methylation patterns and epigenetic memory. Genes Dev.

[32] Laurent L, Wong E, Li G, Huynh T, Tsirigos A, Ong CT, Low HM, Kin Sung KW, Rigoutsos I, Loring J, Wei CL (2010). Dynamic changes in the human methylome during differentiation. Genome Res.

[33] Boots CE, Jungheim ES (2015). Inflammation and human ovarian follicular dynamics. Semin Reprod Med.

[34] Robinson JJ, Ashworth CJ, Rooke JA, Mitchell LM, McEvoy TG (2006). Nutrition and fertility in ruminant livestock. Anim Feed Sci Tech.

[35] Mora JM, Fenwick MA, Castle L, Baithun M, Ryder TA, Mobberley M, Carzaniga R, Franks S, Hardy K (2012). Characterization and significance of adhesion and junction-related proteins in mouse ovarian follicles. Biol Reprod.

[36] Siu MKY, Cheng CY (2012). The blood-follicle barrier (BFB) in disease and in ovarian function. Adv Exp Med Biol.

[37] Blattler A, Yao L, Witt H, Guo Y, Nicolet CM, Berman BP, Farnham PJ (2014). Global loss of DNA methylation uncovers intronic enhancers in genes showing expression changes. Genome Biol.

[38] Goitre L, Balzac F, Degani S, Degan P, Marchi S, Pinton P, Retta SF (2010). KRIT1 regulates the homeostasis of intracellular reactive oxygen species. PLoS One.

[39] Agarwal A, Aponte-Mellado A, Premkumar BJ, Shaman A, Gupta S (2012). The effects of oxidative stress on female reproduction: a review. Reprod Biol Endocrinol.

[40] Zhang Y, Liu T, Meyer CA, Eeckhoute J, Johnson DS, Bernstein BE, Nusbaum C, Myers RM, Brown M, Li W, Liu XS (2008). Model-based analysis of ChIP-Seq (MACS). Genome Biol.

[41] Livak KJ, Schmittgen TD (2001). Analysis of relative gene expression data using real-time quantitative PCR and the 2−ΔΔCT method. Methods.

